# Virtual Reality Objectifies the Diagnosis of Psychiatric Disorders: A Literature Review

**DOI:** 10.3389/fpsyt.2017.00163

**Published:** 2017-09-05

**Authors:** Martine J. van Bennekom, Pelle P. de Koning, Damiaan Denys

**Affiliations:** ^1^Academic Medical Center, Department of Psychiatry, University of Amsterdam, Amsterdam, Netherlands; ^2^The Netherlands Institute for Neuroscience, Royal Netherlands Academy of Arts and Sciences, Amsterdam, Netherlands

**Keywords:** virtual reality, psychiatry, diagnosis, assessment, review

## Abstract

**Background:**

To date, a diagnosis in psychiatry is largely based on a clinical interview and questionnaires. The retrospective and subjective nature of these methods leads to recall and interviewer biases. Therefore, there is a clear need for more objective and standardized assessment methods to support the diagnostic process. The introduction of virtual reality (VR) creates the possibility to simultaneously provoke and measure psychiatric symptoms. Therefore, VR could contribute to the objectivity and reliability in the assessment of psychiatric disorders.

**Objective:**

In this literature review, we will evaluate the assessment of psychiatric disorders by means of VR environments. First, we investigate if these VR environments are capable of simultaneously provoking and measuring psychiatric symptoms. Next, we compare these measures with traditional diagnostic measures.

**Methods:**

We performed a systematic search using PubMed, Embase, and Psycinfo; references of selected articles were checked for eligibility. We identified studies from 1990 to 2016 on VR used in the assessment of psychiatric disorders. Studies were excluded if VR was used for therapeutic purposes, if a different technique was used, or in case of limitation to a non-clinical sample.

**Results:**

A total of 39 studies were included for further analysis. The disorders most frequently studied included schizophrenia (*n* = 15), developmental disorders (*n* = 12), eating disorders (*n* = 3), and anxiety disorders (*n* = 6). In attention-deficit hyperactivity disorder, the most comprehensive measurement was used including several key symptoms of the disorder. Most of the studies, however, concerned the use of VR to assess a single aspect of a psychiatric disorder.

**Discussion:**

In general, nearly all VR environments studied were able to simultaneously provoke and measure psychiatric symptoms. Furthermore, in 14 studies, significant correlations were found between VR measures and traditional diagnostic measures. Relatively small clinical sample sizes were used, impeding definite conclusions. Based on this review, the innovative technique of VR shows potential to contribute to objectivity and reliability in the psychiatric diagnostic process.

## Introduction

In 1973, Dr. David Rosenhan conducted his famous experiment “Being sane in insane places” (1). Eight researchers feigned auditory hallucinations, which led to admission with a psychiatric diagnosis in 12 hospitals in the USA. Nowadays, this study is often quoted to criticize the objectivity and reliability of the diagnostic process in psychiatry. Medical specialists in the somatic field can usually rely on numerous biological markers, such as blood tests and imaging techniques, to confirm and classify the suspected diagnosis. Up till now, such biological markers are lacking in psychiatry. A psychiatric diagnosis is still largely based on a clinical interview, a mental state examination, and multiple questionnaires (2, 3). This imposes the risk of recall bias, since important diagnostic information is dependent upon the memory and current state of mind of the particular patient. Furthermore, there is a risk for interviewer bias since the conclusion on both the diagnosis and the severity of the diagnosed disorder is dependent on the interpretation of the clinician. To support the objectivity and reliability of a psychiatric diagnosis, there is a clear need for new assessment methods. Objective and reliable assessment methods would also contribute to standardization and uniformity for research purposes.

With increasing technological innovation, it is important to consider virtual reality (VR) for this purpose. VR is defined as a computer generated environment using sensory stimuli in which a person can explore and interact (4). The environment can be displayed on a general computer screen, a head-mounted display (HMD), or a surround system. VR environments are generally designed to resemble real life situations and can be extended with specific stimuli to provoke symptoms, like disorder-related items and virtual humans, which are also known as avatars (5). These VR environments, therefore, provide the opportunity to study participants in a lifelike, yet standardized and controlled environment. VR environments are increasingly used in psychiatric therapeutic settings to facilitate virtual exposure in behavioral therapy (6, 7). In this setting, the virtual environment is designed to provoke psychiatric symptoms like fear and stress, after which gradual extinction can follow. If a VR environment is able to provoke and simultaneously measure psychiatric symptoms, it can be used to improve reliability and objectivity in the assessment of psychiatric disorders. The first objective of this literature review is to investigate if VR environments are capable of simultaneously provoking and measuring psychiatric symptoms, thereby showing a relevant difference in outcome measures between patients with a psychiatric disorder and healthy controls. The second objective is to identify correlations between VR outcome measures and traditional diagnostic measures including clinician or patient-rated questionnaires.

## Methods

We searched the databases PubMed, Embase, and Psycinfo from January 1990 until May 2016. We used a combination of the keywords (diagnos*, assess*, or test) and (psychiatry or psychiatric) and VR. This search revealed 360 studies on PubMed. The same keywords revealed an additional two articles in Embase and Psycinfo. Additional searches on multiple psychiatric disorders combined with the terms VR AND diagnos* or assess* were also performed. The studies were screened for title and abstract when the title was suitable. After review, 39 relevant studies were identified. References of these studies and related citations were searched to identify eligible articles not located through the initial search of the electronic databases.

Studies were included if they concerned a VR environment used in the assessment of a psychiatric disorder and made a comparison between a clinical and healthy control sample. We also included studies restricted to a clinical sample investigating a correlation between VR outcome measures and traditional diagnostic measures. We included only English language publications. Studies were excluded if they concerned a VR environment used for therapeutic purposes, restriction to a non-clinical sample, investigation of another technology than VR (e.g., telephone, internet, online modules, videoconferencing, mobile technology, social media), and studies restricted to feasibility testing. Furthermore, studies were excluded if they concerned a computerized version of a neuropsychological test.

## Results

### Psychosis and Schizophrenia

To date, 15 studies have investigated VR environments in the assessment of a psychotic population. Additionally, one review of the implementation of VR in the assessment and treatment of psychotic disorders was found (8). The VR environments measure a certain aspect of psychosis, such as paranoia, social behavior, and cognitive disabilities.

#### Paranoia

Using VR, paranoid ideations in reaction to avatars with a neutral facial expression can be assessed in a standardized environment. This was tested in patients with a psychotic disorder in a virtual underground tube (9, 10), a virtual café, and shopping street (11). In all these studies, paranoid ideations toward the neutral avatars were determined after exposure to the VR environment by scales specifically developed for this purpose, such as the State Social Paranoia Scale (12) and the Virtual Reality Questionnaire (13). The Green et al. Paranoid Thoughts Scale (14) was obtained prior to exposure to the VR environments and used as the traditional measure. Freeman et al. found significantly more paranoid ideations attributed to the neutral avatars in the patient group compared to healthy controls (9). This was in contrast to Fornells-Ambrojo et al. who found no difference between the patient and healthy control groups. However, in the patient group, they found a positive correlation (*r* = 0.62, *p* = 0.004) between paranoid ideation in VR and paranoid ideations in real life (10). Valmaggia et al. also found a positive correlation between rated paranoid thoughts attributed to the avatars and paranoid thoughts experienced in real life (*r* = 0.61, *p* = 0.004) in patients classified as “at risk” for psychosis (15). Furthermore, Veling et al. also found a strong correlation between paranoid ideations in VR and baseline levels of paranoia (*r* = 0.67, *p* < 0.01) (11).

#### Social Behavior

Interestingly, the assessment of social behavior in schizophrenia patients is possible through the interaction with avatars in VR. Dyck et al. showed that schizophrenia patients had more difficulties recognizing displayed emotions on avatar faces compared to healthy controls (16). Kim et al. found lower scores on interpreting avatar emotions, verbal cues, and gestures in patients compared to healthy controls (17). In patients, they found a negative correlation between emotion recognition and positive symptoms (*r* = −0.49, *p* = 0.01) and negative symptoms (*r* = −0.36, *p* = 0.05) as assessed on the positive and negative syndrome scale (PANSS) (18). In other studies, patients showed less gazing toward an avatar during the listening phase of a negative conversation (19), and kept a larger distance toward an avatar compared to healthy controls. Patients with more severe negative symptoms measured through the PANSS kept less distance toward angry avatars (*r* = −0.42, *p* < 0.05) (20).

#### Cognitive Disabilities

Multiple research groups investigated cognitive dysfunction in schizophrenia patients through VR, especially executive functions and memory. Executive functions were assessed through grocery shopping in a virtual supermarket (21), medication management in a virtual apartment (22), and decision making on a bus route (23). In all these studies, a comparison was made between schizophrenia patients and healthy controls. In general, schizophrenia patients showed more erroneous actions in their grocery shopping and medication management, as well as less flexibility and more stress in the decision-making process on the bus routes. Significant correlations were found between scores on subtests of the supermarket environment and subtests on a standardized executive functioning questionnaire (21). The decision time in the bus route task was negatively correlated with negative symptoms determined on the PANSS questionnaire (*r* = −0.76, *p* = 0.01) for schizophrenia patients.

Other research groups investigated both spatial and working memory in a virtual maze (24, 25) and a virtual city (26) in schizophrenia patients compared to healthy controls. In the maze, patients showed a higher error rate, especially in distractor conditions (24). Furthermore, patients needed more time to complete the task and covered more distance in both the maze and the city (25, 26). In patients, negative correlations were found between both time spent on finishing the maze task (*r* = 0.6, *p* < 0.001) and covered distance in the city (*r* = −0.48, *p* < 0.001) and total scores on a neuropsychological battery indicating better short-term memory and spatial learning with better cognitive performance (25, 26). Another research group tested spatial memory on an allocentric and egocentric level (27). Patients differed in a higher number of errors and less learning progress over multiple trials on an allocentric, but not an egocentric level compared to healthy controls.

### Developmental Disorders

So far, four studies have investigated VR environments in the assessment of attention-deficit hyperactivity disorder (ADHD) and eight studies in the assessment of autism.

#### Attention-Deficit Hyperactivity Disorder

In ADHD, the most investigated aspects include errors, distraction, reaction time, and hyperactivity. The research group of Rizzo et al. created a virtual classroom environment in which participants are expected to conduct a task from a traditional continuous performance test (CPT) while distracting visual and auditory stimuli are introduced. The aim was to assess both cognitive functions such as attention and inhibition and physical activity. In multiple studies, a comparison between ADHD patients in the age range of 8–17 years and healthy controls revealed a significantly larger number of commission errors (28, 29), omission errors (29, 30), and a longer reaction time (30) in ADHD patients. Moreover, ADHD patients also showed more errors and an increasing reaction time when the task had to be repeated several times (28). Finally, ADHD patients showed a higher level of overall physical hyperactivity. This was measured by tracking devices attached to the HMD, the hand, and the knee (29). In addition, a significant correlation was found between the percentage of correct answers in VR and a standard CPT (*r* = 0.64, *p* < 0.001) (31) Also, measures of correct hits and hit reaction time in VR and in a standard CPT were significantly correlated (coefficient up to 0.623, *p* < 0.001) (28) as were the number of errors and the amount of hyperactivity in VR and behavioral ratings by parents on a behavior checklist (*r* = 0.51, *r* = 0.59 and *r* = 0.61, respectively) (29).

#### Autism

In autism spectrum disorder (ASD), some studies in this field are directed at the assessment of social behavior in a virtual environment. Emotion recognition, social distance, empathy, and socially appropriate behavior were evaluated. Emotion recognition and empathy in response to avatar faces showing basic emotions were tested in two studies (32, 33). Both studies found the ASD and healthy control groups were equally able to correctly recognize the displayed emotions; however, the ASD groups took more time in selecting the correct emotion and showed less confidence afterwards (32). Furthermore, the ASD group showed less congruent emotions in response to the emotion displayed by the avatar (33). Parsons et al. tested appropriate social behavior in a virtual bar (34). Compared to healthy controls, the ASD patients showed less of a tendency to avoid a couple standing at an otherwise empty bar when ordering drinks. Kim et al. found subjects with ASD were more likely to virtually distance themselves by using a joystick from a “happy” approaching avatar compared to healthy controls despite correctly identifying the emotion of the avatar (35).

Other studies in this field are directed at gaining insight in the pathophysiology of ASD by studying neural mechanisms of deviant non-verbal behavior (36). Four research groups investigated the neural mechanisms in response to avatar gaze direction or duration in ASD subjects (37–40). They consistently found deviations in the networks involved in the theory of mind (ToM) and gaze perception in ASD subjects compared to healthy controls. Von Dem Hagen et al. found an increased activation in both the ToM and gaze perception networks in response to direct gaze in healthy control and an activation of the same networks in response to averted gaze in ASD subjects (40). Schulte-Rüther et al. investigated the neural processing of emotional responses to an emotion displayed by an avatar in ASD subjects. They found several deviations, which included an increased activation of the right temporoparietal junction and the dorsal area of the medial prefrontal cortex during processing in ASD subjects, speculating subjects with ASD address different cognitive strategies to access their own emotional state in response to emotions displayed by others (33).

### Forensic Psychiatry

Only one study evaluated the use of VR in the assessment of forensic patients. The research group of Renaud et al. tested both sexual arousal and oculomotoric response in reaction to virtual adult and child avatars that were naked in child molesters and healthy controls. They found a more pronounced erectile response in child molesters in reaction to the child avatars, as opposed to healthy controls who showed this response to the adult avatars. Also, a more focused gaze toward the sexual relevant areas in all avatars was found in the child molester group, possibly referring to their sexual preoccupation (41).

### Substance Use Disorders

In substance use disorders, VR is mainly used to study craving (42). Craving is an important diagnostic criterion of substance use disorders and is defined as an intense preoccupation or urge to use the desired substance (43). Most of the studies using VR to assess craving in alcohol-, cannabis-, and hard drug dependence focused on a comparison between neutral and substance cue-related VR environments in patients with a substance use disorder and did not include a healthy control group.

Virtual reality was used to create realistic scenes including substance-related cues, avatars, and even the smell of beer or cannabis. The studies generally showed a higher level of subjective craving in VR environments containing substance-related cues compared to neutral VR environments (44–48). Only in the study of Saladin et al., physiological measurements were included; they found an increased heart rate in the cue-related environment compared to the neutral VR environment in cocaine-dependent patients (48).

Finally, in a study by Lee et al., a comparison was made between alcohol-dependent patients and healthy controls (49). They tested a VR environment with alcohol-related cues and an environment with avatars exerting social pressure. Their results showed that both patients and healthy controls experienced an increase in subjective craving in response to virtual social pressure in the absence of alcohol related cues. However, only the alcohol-dependent patients experienced an increase in craving in response to the VR environment with alcohol-related cues, as opposed to the healthy controls.

### Eating Disorders

We found three studies using VR in the assessment of eating disorders, additionally, a review article was identified (50). The studies focused on body image and body dissatisfaction, but also on anxiety and physical reactions in response to virtual food or dining scenarios.

Gorini et al. investigated anxiety and physical reactions in response to virtual food (51). In eating disorder patients, anxiety, heart rate, and skin conductance in response to virtual food were significantly higher compared to healthy controls. Ferrer-García et al. studied body-image, body-dissatisfaction, and depressive and anxious symptoms at confrontation with virtual dining scenarios in eating disorder patients and healthy controls (52). Patients showed more body distortion and dissatisfaction in all virtual scenarios and a significantly higher level of anxiety and depression compared to healthy controls, especially in the scenarios with high calorie food. The same research group examined food craving in response to virtual food in patients with bulimia and binge eating disorder compared to healthy controls. They found a higher level of subjective food craving, determined by a visual analog scale (VAS), in the patient group in response to the virtual food compared to healthy controls. They suggest this could underlie the binges and higher BMI in patients with bulimia and binge eating disorder (53).

### Mood and Anxiety Disorders

We found just a solitary study on the use of VR in the assessment of mood disorders; Gould et al. studied the spatial memory of patients with a depressive disorder in a virtual city were different locations had to be located (54). Depressive patients found significant fewer locations in the virtual city, and this finding was correlated with the Montgomery–Asberg Depression Rating Scale (55) (*r* = −0.33, *p* = 0.02) and the performance on a spatial working memory task (*r* = −0.45, *p* = 0.007).

In anxiety disorders, six studies were identified. We found four studies on self-reported and physiological anxiety responses to a virtual tunnel in patients with tunnel phobia (56), to virtual combat scenes in veterans with PTSD (57), in a virtual bus trip in patients with panic disorder (58), and in response to a virtual audience in social anxiety (59). In the tunnel phobic patients group, self-reported anxiety was measured by means of the subjective unit of discomfort scale (SUDS) (60). Both the self-reported anxiety and heart rate increased with the transition of an open to a tunnel VR-environment. The skin conductance level was higher compared to healthy controls in all environments (56). The veterans with PTSD showed a slower decrease in skin conductance amplitude compared to healthy controls in the course of five virtual combat scenes, possibly indicating slower habituation. No correlations between the physiological measures and the self-reported anxiety and PTSD-related questionnaires were determined (57). During a virtual bus trip, patients with panic disorder had higher scores on self-reported anxiety (SUDS), panic attack symptoms (diagnostic symptom questionnaire) (61), and physiological anxiety measures compared to healthy controls (58). Cornwell and colleagues investigated patients with social anxiety in a virtual public speech setting by means of startle reactivity as a physiological anxiety measure in addition to self-reported levels of distress on a VAS. They found significantly higher self-reported anxiety and startle reactivity in response to focused attention of the audience (59). In all these studies, the physiological anxiety measures are heterogeneous but generally in line with the self-reported anxiety measures.

Finally, the research group of Kim et al. developed a virtual house and office for the assessment of both anxiety and compulsions in obsessive–compulsive disorder (OCD) (62, 63). They found a higher level of provoked anxiety (VAS) before and after checking a situation in OCD patients compared to healthy controls, and a larger decrease in anxiety after checking. Furthermore, a correlation of VR-anxiety with the Yale–Brown Obsessive–Compulsive Scale (Y-BOCS) (64) and Beck’s Anxiety Inventory (65) was found (*r* = 0.35, *p* < 0.05 and *r* = 0.45, *p* < 0.01). They also found OCD patients showed a higher checking frequency, spent more time on checking, a longer trajectory in the environment, and more gazing time during checking behavior. The Y-BOCS was correlated with both gazing time (*r* = 0.48, *p* < 0.05) and checking time (*r* = 0.51, *p* < 0.05).

## Discussion

The primary purpose of this review was to investigate whether VR environments used in the assessment of psychiatric disorders were able to show a relevant difference in outcome measures between patients with a psychiatric disorder and healthy controls. Generally, the virtual environments were able to show significant differences in outcome measures between patients and healthy controls. This highlights the great potential of VR in a psychiatric assessment. Only in two studies on emotion recognition in autism and paranoia in psychosis (10, 32), no significant differences were found between patients and healthy controls, probably due to small sample sizes or clinical groups with relatively mild symptoms.

The results on the use of VR in the assessment of psychotic disorders show virtual environments are capable of provoking paranoid ideations and assess deficits in social behavior and cognitive disabilities in patients. This shows great potential for the assessment of a population that might be more at ease with a computerized assessment than a face-to-face assessment with a clinician. From the studies on VR in ADHD and autism, it is clear that the inclusion of parameters such as hyperactivity measured by tracking devices and neural mechanisms measured by fMRI is of value. If a virtual environment is able to activate both subjective symptoms and the neural and physiological substrate associated with a disorder, this can contribute to a more comprehensive and objective assessment of this disorder. For children with ADHD and autism, the implementation of VR to assess symptoms would provide a more realistic and immersive experience than current laboratory settings, possibly contributing to increased motivation and participation. In forensic psychiatry, VR provides the opportunity to expose offenders with a psychiatric disorder to potential risky situations in order to evaluate their symptoms, without posing an actual threat to the environment (66). Therefore, although research in this area is scarce, forensic psychiatry has great potential for VR assessment. In substance use disorders pertaining the misuse of alcohol- and cannabis, VR seems to be an adequate technique to provoke craving. The addition of olfactory stimuli complements the experience in VR. However, studies on other substances including hard drugs are necessary before definite conclusions can be drawn. In eating disorders, VR has the potential to assess both key symptoms like a deviant body image and additional symptoms like anxiety. In these studies, only virtual food or dining scenarios were used to provoke symptoms. It would be interesting to explore more possibilities, for example, by assessing body image in relation to avatars with various body shapes. The use of VR in anxiety disorders is promising since physiological mechanisms of the anxiety response are largely known. This enables the inclusion of physiological measures such as heart rate, respiration rate, and skin conductance in response to a virtual environment, thus contributing to an objective assessment of anxiety (67).

Efforts are made to create VR environments to assess multiple symptoms of a disorder; the study of Rizzo evaluated the most comprehensive VR environment, evaluating both cognitive functions like attention and distractibility and physical activity, which are core symptoms of ADHD (28–31). However, in most of the reviewed studies, VR was used to assess only one symptom of the specific disorder. Interestingly, this could be in line with the introduction of dimensional criteria to the categorical diagnostic classification in the fifth edition of the Diagnostic and Statistical Manual of Mental Disorders (68). In this edition, apart from marking a symptom as present, dimensions are included to determine symptom severity. It would be worthwhile to investigate if VR can be used to measure the severity of specific symptoms transdiagnostically. More research on the correlation between traditional symptom severity measures and VR environments in patients in different diagnostic categories would be necessary to explore this potential.

The second purpose was to determine correlations between VR outcome measures and traditional diagnostic measures in patients with psychiatric disorders. Not all studies included a correlation measurement. In 14 studies, significant correlations were found between VR outcome measures and traditional measures. The authors usually expressed a preference for VR because contrary to retrospective traditional measures, in VR, real-time assessments of behavior, affect, and social interaction are possible in response to realistic environments resembling daily activities (69). However, VR outcome measures were often determined using symptom scoring through both state and trait questionnaires after immersion in the VR environment. State questionnaires are designed to measure temporarily induced symptoms whereas trait questionnaires are designed to measure an enduring disposition (70). State questionnaires, like the State Social Paranoia Scale, therefore, seem more suited for this purpose. It could be argued that short state questionnaires obtained during instead of after the VR experience are more objective, though this might disturb the immersion in the virtual environment. The objectivity of a VR measurement can be further improved when other measures are added, such as physical activity, eye tracking, distance tracking, physiological measures, and neural activation. These measures can often be obtained without distracting the participants so the immersion in the VR environment is not disturbed. The addition of these measures contributes to a more objective assessment of the psychiatric disorder.

Since this literature review concerns a novel technique, which has only recently been investigated in the psychiatric diagnostic process, sample sizes of the included studies are generally small. Furthermore, only publications in the English language have been included in this review. Due to the great variety in investigated virtual environments and outcome measures, merging the results was not possible. More studies with larger sample sizes and more uniformity in outcome measures are necessary to enable a more systematic review including a meta-analysis. Not all studies reported results on correlations between the VR-measures and traditional measures of the aspect of the disorder, presumably because of small sample sizes or absence of comparable assessment methods. This impairs a conclusion on construct validity.

In conclusion, VR shows potential to be implemented in the diagnostic process. The advantages are numerous and include the use of an immersive, modern technique instead of a standard laboratory setting that could motivate participating patients. Most of the studies investigated made use of computer-animated VR environments with divergent levels of realism. New VR techniques such as video VR or augmented reality are developing at great speed and will drastically improve the resemblance to real-life situations. This will enlarge the efficacy of virtual environments in assessing psychiatric disorders. Furthermore, standardized environments can be created in VR to ensure uniformity if the VR is used for research purposes. Finally, the resemblance to real life situations creates enormous opportunities to assess patients in enclosed settings, for example, patients in detention.

## Author Contributions

MB, PK, and DD contributed to the conception of this manuscript. MB acquainted and interpreted the data for this manuscript. MB drafted this manuscript, which was critically revised for important intellectual content by PK and DD. All authors approved the submitted version of this manuscript. All authors agree to be accountable for all aspects of the work.

## Conflict of Interest Statement

The authors declare that the research was conducted in the absence of any commercial or financial relationships that could be construed as a potential conflict of interest.
